# Dichotomal functions of phosphorylated and unphosphorylated STAT1 in hepatocellular carcinoma

**DOI:** 10.1007/s00109-018-1717-7

**Published:** 2018-11-19

**Authors:** Buyun Ma, Kan Chen, Pengyu Liu, Meng Li, Jiaye Liu, Kostandinos Sideras, Dave Sprengers, Katharina Biermann, Wenshi Wang, Jan N. M. IJzermans, Wanlu Cao, Jaap Kwekkeboom, Maikel P. Peppelenbosch, Qiuwei Pan

**Affiliations:** 1 Department of Gastroenterology and Hepatology, Erasmus MC-University Medical Center, Room Na-617, ‘s-Gravendijkwal 230, 3015CE Rotterdam, The Netherlands; 2 College of Life Sciences, Zhejiang Sci-Tech University, Hangzhou, 310018 China; 3 Department of Pathology, Erasmus MC-University Medical Center, Rotterdam, 3015CE The Netherlands; 4 Department of Surgery, Erasmus MC-University Medical Centre, Rotterdam, 3015CE The Netherlands

**Keywords:** Hepatocellular carcinoma (HCC), Signal transducer and activator of transcription 1 (STAT1), Interferon (IFN) signaling, Immune response

## Abstract

**Abstract:**

Interferons (IFNs) with antiviral and immune-stimulatory functions have been widely used in prevention and treatment of hepatocellular carcinoma (HCC). Signal transducer and activator of transcription 1 (STAT1) is a key element of the IFN signaling, and the function of STAT1 is critically determined by its phosphorylation state. This study aims to understand the functions of phosphorylated (p-) and unphosphorylated (u-) STAT1 in HCC. We found that u-STAT1 is significantly elevated in patient HCC tumor tissues and predominantly expressed in cytoplasm; while p-STAT1 is absent. Loss of u-STAT1 potently arrested cell cycle and inhibited cell growth in HCC cells. Induction of p-STAT1 by IFN-α treatment effectively triggers the expression of interferon-stimulated genes (ISGs), but has moderate effect on HCC cell growth. Interestingly, both u-STAT1 and p-STAT1 are induced by IFN-α, through with distinct time-dependent process. Furthermore, the ISG induction patterns mediated by p-STAT1 and u-STAT1 are also distinct. Importantly, artificial blocking of the induction of u-STAT1, but not p-STAT1, sensitizes HCC cells to treatment of IFNs. Therefore, p-STAT1 and u-STAT1 exert dichotomal functions and coordinately regulate the responsiveness to IFN treatment in HCC.

**Key Messages:**

- STAT1 is upregulated and predominantly presented as u-STAT1 in HCC, while p-STAT1 is absent.
- U-STAT1 sustains but p-STAT1 inhibits HCC growth.
- The dynamic change of phosphorylation state of STAT1 control the responsiveness to IFN treatment.

**Electronic supplementary material:**

The online version of this article (10.1007/s00109-018-1717-7) contains supplementary material, which is available to authorized users.

## Introduction

Hepatocellular carcinoma (HCC) is one of the most common malignant tumors [1] and the second leading cause of cancer-related death worldwide [2]. As a major etiology, chronic infection with hepatitis B or C virus (HBV or HCV) triggers liver fibrosis, cirrhosis, and eventually the development of HCC [3]. To prevent from or treat for viral hepatitis-related HCC, interferons (IFNs) have been explored in clinic [4, 5]. In context of tumors, IFNs can be produced by various cell types, including immune cells, as well as tumor cells. They elicit antitumor effects by directly controlling tumor cells or indirectly by regulating immune response [6]. On the contrary, the pro-tumorigenic effect of IFNs, which may help the tumor escape the recognition of the immune system through “immunoediting,” has also been reported [7, 8]. However, which side wins the tussle appears to be dependent on the context of tumor type, microenvironmental factors, and signaling intensity [7]. But the exact mechanisms remain poorly understood due to their multitude functions in respect to both intra-tumoral and micro-environmental determinants [9]. Although benefits of reducing cancer risk have been observed in clinical studies [5], IFN treatment for the management of HCC is still controversial and no clear recommendations have been proposed [9].

Signal transducer and activator of transcription 1 (STAT1), an important upstream regulator of the IFN signaling, functions as the core transcription factor to drive the transcription of a subset of IFN-regulated genes (IRGs) [10]. Upon IFN stimulation, phospho-STAT1 (p-STAT1) acts as a key element for STAT1 homodimerization (STAT1-STAT1) or heterodimerization (STAT1-STAT2-IRF9 complex, ISGF3). These complexes translocate to the nuclear with subsequent binding to interferon-stimulated response elements (ISRE) and interferon-gamma activated sequences (GAS), and then stimulate the transcription of IRGs to regulate host immune response and cell growth [11]. Although STAT1 has been found to be deregulated in a variety of cancers, the exact role of STAT1 in cancer, especially in different types of cells, remains controversial. On one hand, STAT1 is recognized as a tumor suppressor which can inhibit tumor growth through regulating cell proliferation, differentiation, and death [12–15]. On the other hand, STAT1 can also be a tumor promoter as it can promote tumor cell growth, therapy resistance, and immune suppression [16, 17]. In addition, expression of STAT1 has been found to correlate with both good and poor prognosis in different types of cancers [8]. Although STAT1 was reported to be a potential suppressor in HCC [18], the findings are based on limited numbers of patients and a modest effect on HCC cell growth.

Upon IFN stimulation, p-STAT1 and unphospho-STAT1 (u-STAT1) act as two forms of STAT1 to perform its function [19]. Although p-STAT1 is recognized as the key activator of IFN signaling, u-STAT1 can also regulate gene transcription in the absence of IFN stimulation [20]. Thus, p-STAT1 and u-STAT1 stimulate transcription of different subsets of genes, which have distinct functions in immune responses of tumors to IFN-related therapy [9]. A subset of ISGs with pro-apoptotic and anti-proliferative functions have been identified to be stimulated by STAT1 during IFN treatment, through which regulate tumor growth [21–23]. Furthermore, immune effector cells can also be activated by IFNs and recruited into tissues, which may augment the anti-tumor effects by inducing apoptosis of the target cells. In contrast, chronic exposure of cells to low IFNs under pathological conditions may steadily induce expression of ISGs that are controlled by u-STAT1, denoted as IFN-related DNA damage resistance signature (IRDS), which can promote tumor growth and metastasis [24]. Therefore, p-STAT1 and u-STAT1 were thought to have distinct functions and have been used as independent prognostic markers in predicting disease outcomes in cancer [25].

In this study, we investigated the expression and functions of p-STAT1 and u-STAT1 in HCC. Remarkably, we found that STAT1 was predominantly present as u-STAT1 form and was highly expressed in the cytoplasm of tumor cells from HCC patients. Although p-STAT1 induced by IFN-α treatment robustly stimulated ISG expression by activating the IFN signaling pathway and inhibited HCC growth, its function was quickly blocked by intrinsic or induced u-STAT1. Thus, the tumor-suppressive or tumor-promoting role of STAT1 largely depends on its phosphorylation status. The dynamic induction of p-STAT1 and u-STAT1 by IFN treatment coordinately regulates the growth of tumor cells.

## Material and methods

### Tissue microarray

Archived formalin fixed paraffin-embedded tissue samples from 133 patients who underwent hepatic resection for HCC at Erasmus MC-University Medical Center between 2004 and 2014 were used for this study. Clinical data of this HCC cohort have been published previously [26]. The use of patient materials was approved by the medical ethical committee of Erasmus MC. Tissue microarray (TMA) slides contained three or four 0.6-mm cores from the tumorous area and two 0.6-mm cores from the paired tumor-free liver (TFL) area of these patients.

### Quantitative real-time polymerase chain reaction

Cells were washed with PBS for two times in indicated time and cell lysates were prepared. RNA was isolated using a Machery-NucleoSpin RNA II kit (Bioke, Leiden, Netherlands) and quantified using a Nanodrop ND-1000 (Wilmington, DE, USA). RNA (500 ng) was employed to make cDNA using a cDNA Synthesis Kit (Takara BIO INC). SYBRGreen-based real-time PCR (MJ Research Opticon, Hercules, CA, USA) was used to amplify the target genes. The program was set as 95 °C for 10 min; 40 cycles at 95 °C for 15 s and at 58 °C for 30 s according to the manufacturer’s instructions. GAPDH was considered as reference gene to normalize target gene expression. Human primer sequences used for qPCR are included in Table S1.

### Statistical analysis

Statistical analysis was performed by using the nonparametric Mann–Whitney test for paired or non-paired data, or the paired *t* test using GraphPad InStat software as appropriate. Crude (non-adjustment) survival analysis (Kaplan-Meier curve) was first used to display the overall survival difference. Hazard ratios (HRs) and 95% Cls were calculated to evaluate the prognostic power of variables of patients. *P* value < 0.05 was considered statistically significant.

## Results

### STAT1 expression is elevated in tumor tissues of hepatocellular carcinoma patients

**Fig. 1:**
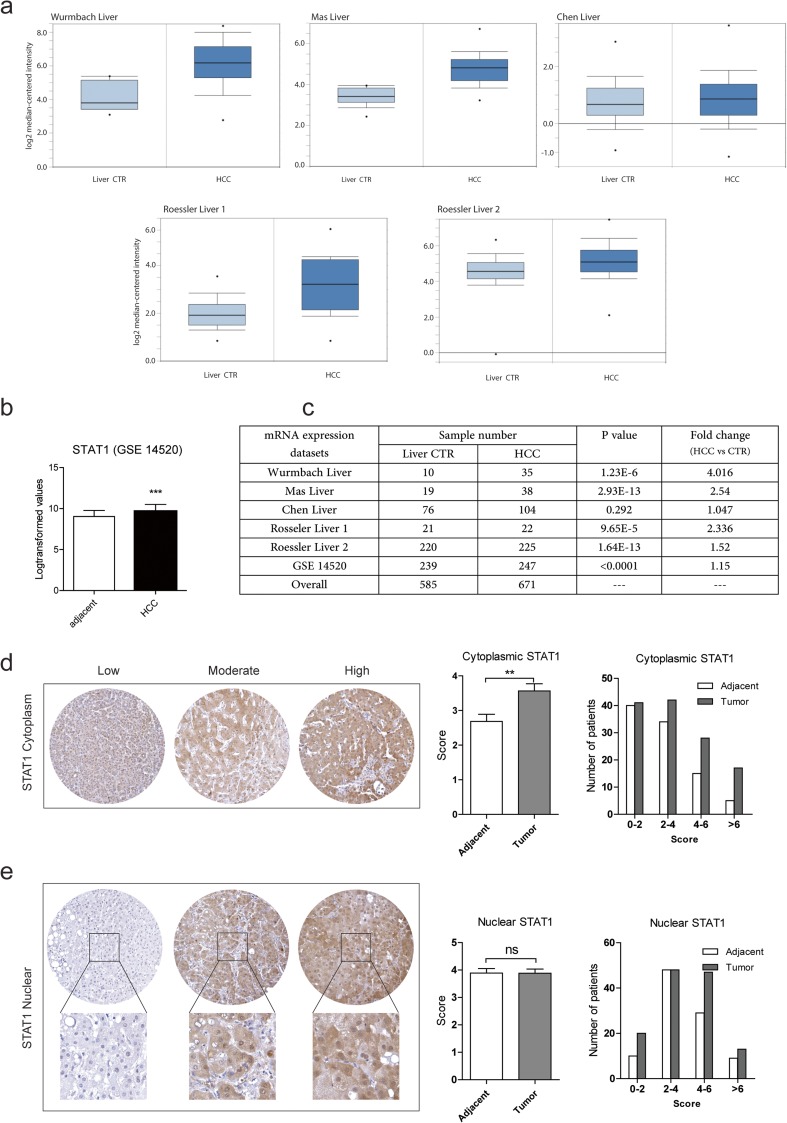
STAT1 expression is upregulated in tumors of HCC patients. **a** The Oncomine microarray database (https://www.oncomine.org) was searched to analyze mRNA expression of STAT1 in HCC patients. In total, five cohorts of 424 HCC tumor tissues compared with 346 paired tumor-free tissues from the same patients were identified. STAT1 mRNA was significantly upregulated in tumor tissues compared with tumor-free tissues in four of the five cohorts, *P* < 0.001. **b** STAT1 expression profile across 486 patient samples including 247 HCC tumors and 239 tumor-free liver tissues (including 186 paired samples) derived from GEO Datasets (GSE 14520). STAT1 mRNA expression was significantly higher in tumor tissues comparing with tumor-free tissues (mean ± SD, ***P < 0.001). **c** Landscape of all the online cohorts. **d** Cytoplasmic STAT1 was significantly upregulated in HCC tumors. The cytoplasmic STAT1 protein immune-reactivity scores (IRS), obtained by multiplying the scores for proportions of stained cells and the scores for expression intensity, range from low (score 0–3), moderate (score 3–6), and high (score 6–9) (mean ± SEM, *n* = 133, **P < 0.01). **d** No significant difference was found in nuclear STAT1 expression IRS scores (mean ± SEM, *n* = 133, ns, no significant)

In order to investigate STAT1 expression in HCC patients, we first searched the online datasets from Oncomine and GEO Datasets (GSE 14520), including six cohorts of 671 HCC tumor tissues with 585 tumor-free liver tissues. To our surprise, STAT1 mRNA expression was significantly upregulated in tumors of five of the six cohorts (Fig. 1a–c). To further confirm these results, TMA slides including tumor tissues and paired tumor-free liver tissues of 133 HCC patients were stained for STAT1. Positive staining of STAT1 in both nuclear and cytoplasm was found in most of the patients. Nuclear STAT1 is often recognized as p-STAT1, while cytoplasmic STAT1 is referred as u-STAT1 [27]. Therefore, we scored the nuclear and cytoplasm expression of STAT1 separately. Consistent with the RNA expression data derived from the online datasets, cytoplasmic STAT1 protein expression in tumor tissues was significantly higher than that in tumor-free tissues (Fig. 1d), but no difference in nuclear STAT1 expression between tumors and tumor-free tissues was found (Fig. 1e).

Among all the clinical factors, alpha-fetoprotein (AFP) serum level and tumor differentiation were significantly associated with higher patient mortality (Table S2). This result is consistent with the general consensus that serum AFP is an independent indicator for HCC prognosis [28]. Correlation of STAT1 expression with clinical behavior were further analyzed. High cytoplasmic STAT1 was not significantly associated with the analyzed factors (Table S3). However, high nuclear STAT1 expression was significantly associated with patient age (Table S4). Furthermore, no significant correlation was observed between STAT1 expression and patient survival outcome (Fig. S1). Collectively, we found that cytoplasmic STAT1 expression in tumor tissues appears higher compared to tumor-free tissues.

### p-STAT1 is absent in tumor tissues of hepatocellular carcinoma patients and human hepatoma cell lines

As the key component of JAK-STAT signaling, STAT1 is phosphorylated after activation and then translocates to the nucleus. Although u-STAT1 has been generally recognized as present in cytoplasm, emerging evidence has indicated its translocation to nuclei and its function as a transcription factor [29].

**Fig. 2:**
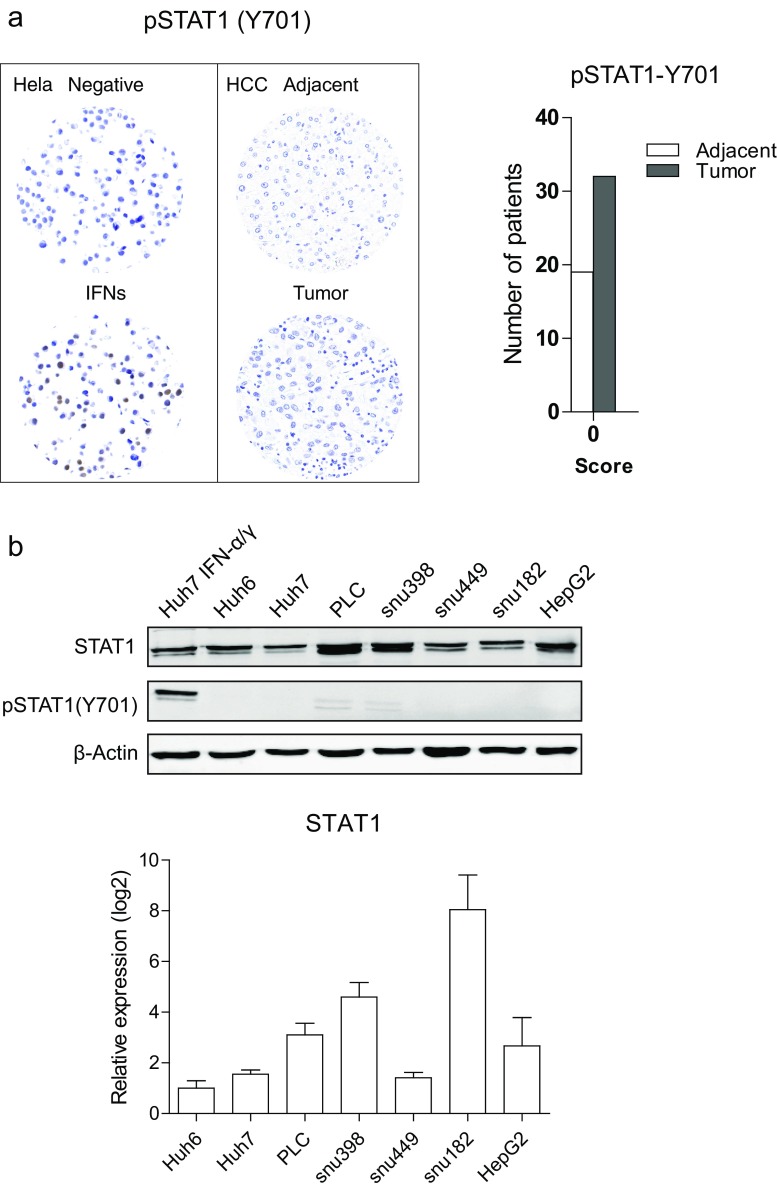
p-STAT1 is absent in both HCC tumors and cell lines. **a** No expression of p-STAT1 in HCC tumors and tumor-free tissues. Tumors (*n* = 32) and tumor-free tissues (*n* = 19) were stained for p-STAT1 (Y701). Paraffin-embedded Hela cells treated with IFNs were used as a positive control. **b** Absence of p-STAT1 in HCC cell lines. Cell lysates were collected for Western blot, and qRT-PCR was used to measure the mRNA levels of STAT1 (mean ± SD, *n* = 3 independent experiments, two biological repeats for each)

To clarify the phosphorylation status and localization of STAT1, we stained TMA slides with tissues of 32 patients with specific antibodies against phosphorylated STAT1. Hela cells treated with IFNs were used as a positive control. Surprisingly, we did not observe positive staining for p-STAT1 in both tumor and tumor-free tissues (Fig. 2a). Consistently, p-STAT1 was absent in all HCC cell lines, whereas u-STAT1 was highly expressed (Fig. 2b). Thus, we have demonstrated that STAT1 is predominantly present in unphosphorylated state in HCC tissues and human hepatoma cell lines.

### Knockout of u-STAT1 impairs hepatocellular carcinoma cell growth

**Fig. 3:**
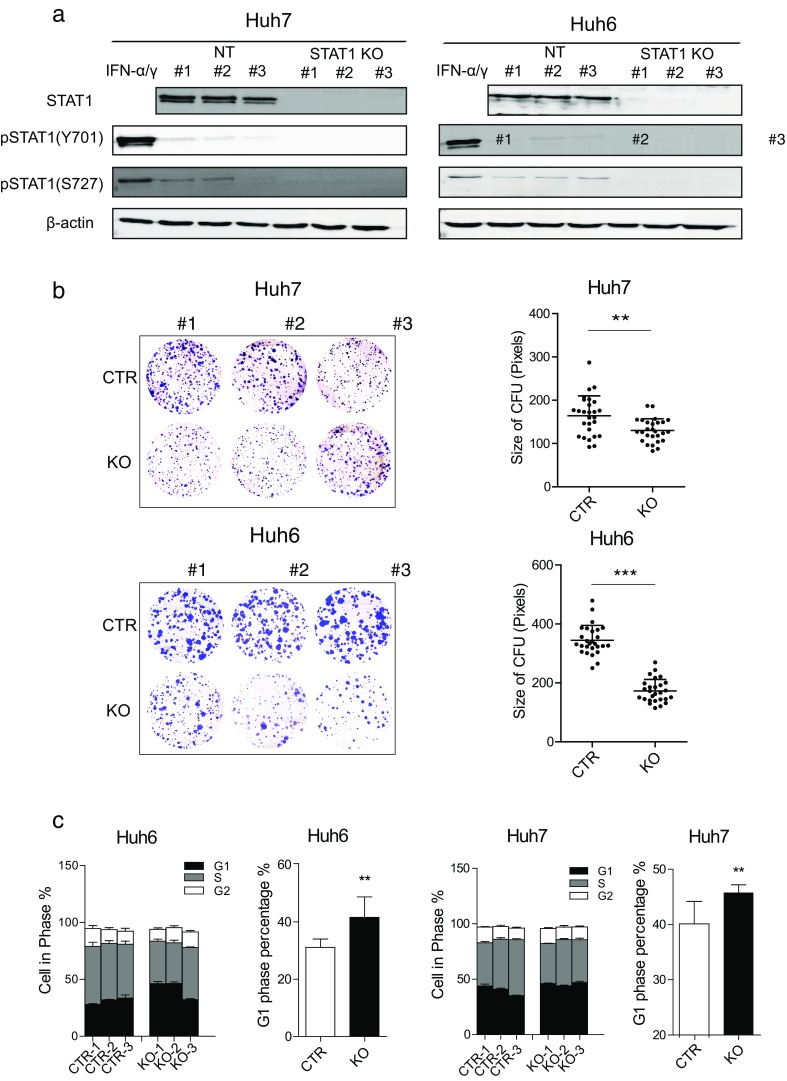
Knockout of u-STAT1 inhibits HCC cell growth. **a** Knockout of STAT1 in HCC cell lines. Cell lysates from Huh7 and Huh6 clones transduced with LentiCRISPR/Cas9 vector were collected for western blot. β-actin served as loading control. **b** U-STAT1 knockout significantly inhibited the colony formation of Huh7 and Huh6 cell lines, as measured by clone size (mean ± SD, *n* = 27, ***P < 0.001; **P < 0.01). **c** U-STAT1 knockout arrested cell cycling. U-STAT1 knockout arrested Huh7 and Huh6 cells in G1 phase determined by flow cytometric analysis (mean ± SD, *n* = 9. **P < 0.01)

To determine the functions of u-STAT1, we generated u-STAT1 knockout cells by Lenti-CRISPR/Cas9 system in Huh7 and Huh6 HCC cell lines (Fig. S2). Complete loss of STAT1 was demonstrated at protein level by western blot analysis (Fig. 3a). Finally, three wild-type and three knockout clones of both cell lines were selected for subsequent experimentation. The colony formation unit (CFU) assay measures the ability of single cells to form clones. Strikingly, we observed that knockout of u-STAT1 inhibited CFU formation of HCC cells (Fig. 3b), in contrast to previous findings that STAT1 served as a tumor suppressor [18, 25]. Cell cycle analysis revealed that loss of u-STAT1 significantly increased the proportion of Huh7 and Huh6 cells in the G1 phase and concomitantly decreased the proportion of cells in S-phase (Fig. 3c). These data suggest that u-STAT1 sustains HCC cell growth.

### Activation of STAT1 phosphorylation by IFN-α treatment hardly inhibits hepatocellular carcinoma cell growth

**Fig. 4:**
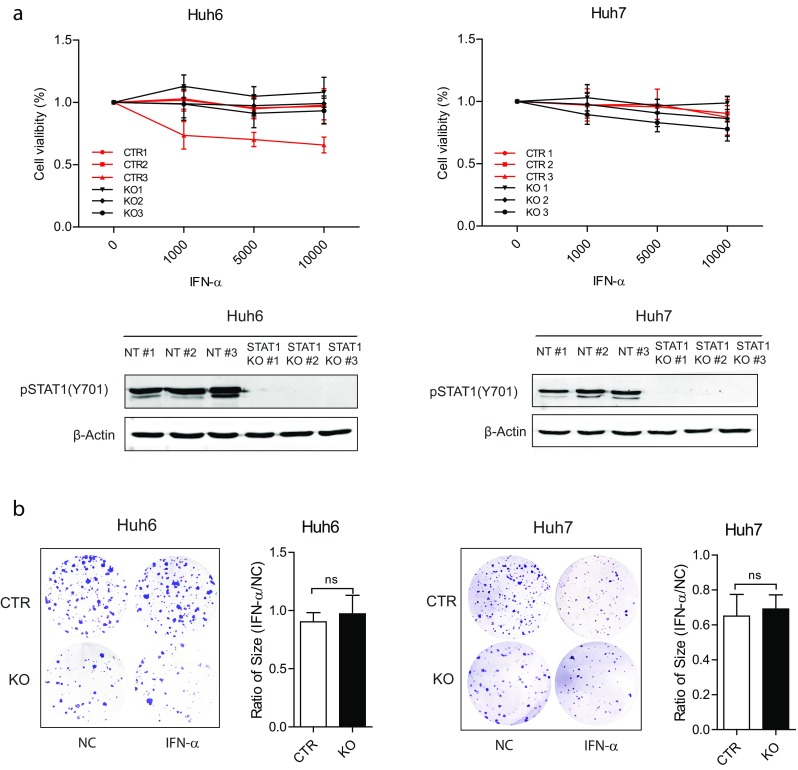
IFN-α exerts modest inhibition on HCC cells independent of p-STAT1. **a** IFN-α treatment did not or only modestly inhibit HCC cell growth independent of p-STAT1. Huh7 and Huh6 cells were treated by IFN-α (1000, 5000 and 10,000 IU/ml) for 7 days, and cell growth was determined by MTT assay (mean ± SD, *n* = 4 independent experiments with triplicates for each). p-STAT1 was measured by western blot and was strongly stimulated by treatment of IFN-α (1000 IU/ml) for 30 min. **b** IFN-α (1000 IU/ml) modestly inhibited the colony formation. Clone size of IFN-α untreated HCC cells were normalized to treated cells, and data were present as STAT1 KO cells comparing with controls (CTR) (mean ± SD, *n* = 3, ns, not significant)

As the active form of STAT1, p-STAT1 has been widely recognized as the functional form in inhibiting tumor growth through inducing cell apoptosis and regulating cell cycle [8, 21]. Because p-STAT1 is absent in HCC cells, IFN-α was employed to activate STAT1 phosphorylation. Upon IFN-α treatment, p-STAT1 was strongly induced in Huh7 and Huh6 cells, but not in STAT1 knockout cells (Fig. 4a). Huh7 and Huh6 with or without STAT1 were treated with different concentrations of IFN-α. Surprisingly, both cells lines were resistant to IFN-α treatment on cell proliferation, although Huh7 cells showed modest growth inhibition. Furthermore, no significant difference of cell growth between STAT1 knockout cells and WT controls was observed (Fig. 4a). Consistent to the MTT results, only Huh7 cells showed a slight inhibition on colony formation and no difference was found between knockout and WT cells in both cell lines (Fig. 4b).

**Fig. 5:**
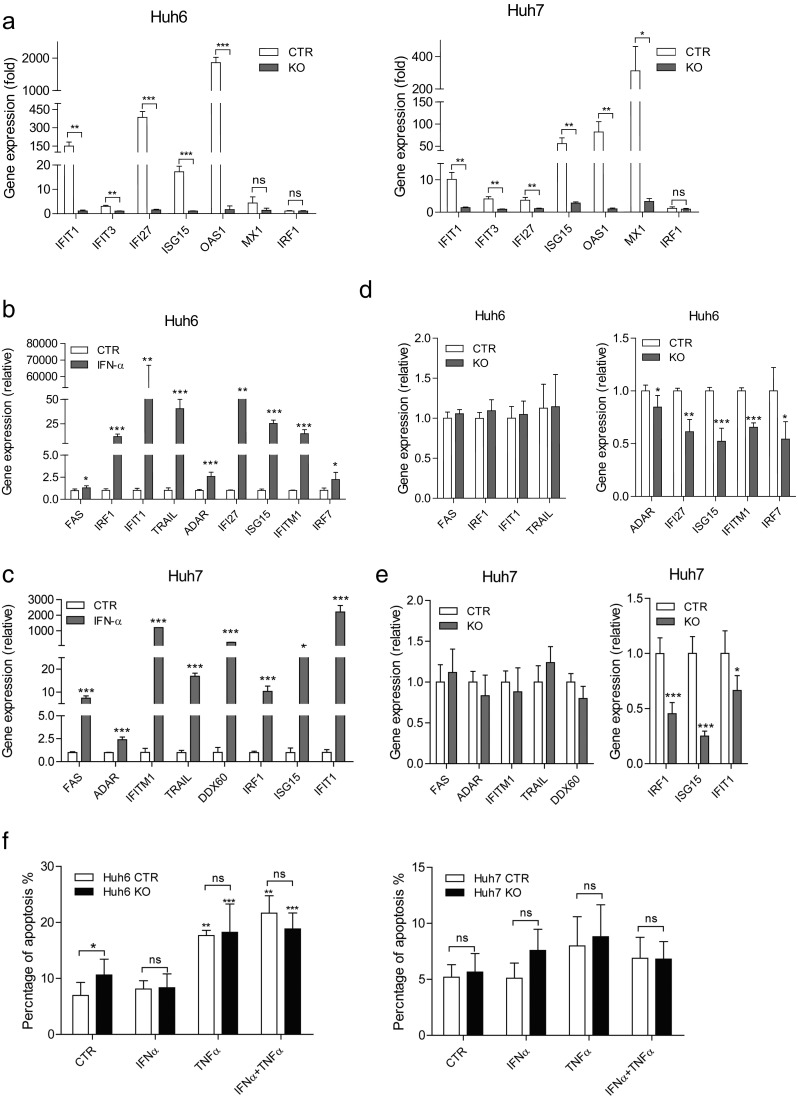
STAT1 is the key component for IFN-α-induced ISG expression but not for cell apoptosis. **a** STAT1 knockout abolished the induction of ISGs by IFN-α. Huh6 KO and Huh7 KO cells were treated with IFN-α (1000 IU/ml) for 24 h. ISG expression was determined by qRT-PCR (mean ± SD, *n* = 3, two biological replicates for each independent experiment, *P < 0.05; **P < 0.01; ***P < 0.001). **b**, **c** p-STAT1 strongly induced the expression of different ISGs. Huh6 and huh7 cells were treated with IFN-α for 4 h. ISGs were quantified by qRT-PCR (mean ± SD, *n* = 4, *P < 0.05; **P < 0.01; ***P < 0.001). **c**, **d** u-STAT1 regulates IRDS genes but not pro-apoptotic ISGs. The expression of ISGs was compared between control and knockout cells in huh6 and huh7 by qRT-PCR (mean ± SD, *n* = 4, *P < 0.05; **P < 0.01; ***P < 0.001). **f** HCC cell lines are resistant to apoptosis induction by treatment of IFN-α. Huh6 KO and Huh7 KO cells were treated with IFN-α (1000 IU/ml), TNF-α (20 ng/ml), or the combination for 72 h. Cells were collected and stained with Anexin V/PI, and subsequently analyzed by FACS (mean ± SD, *n* ≥ 4, *P < 0.05, ns, not significant)

Induction of ISGs is the hallmark of STAT1 activation [30]. As expected, a subset of ISGs were strongly induced by IFN-α treatment, while the stimulation was abolished in STAT1 knockout cells (Fig. 5a). Besides, the difference of gene regulations between p-STAT1 and u-STAT1 were further compared. ISGs with pro-apoptotic functions, such as FAS, TRAIL, were found only regulated by p-STAT1 during IFN stimulation (Fig. 5b, c). ISGs of the IRDS members that are known to promote tumor growth and metastasis are regulated by u-STAT1 (Fig. 5d, e). However, we failed to observe the apoptosis induction of IFN-α in HCC cells, while TNF-α did in Huh6 cells (Fig. 5f). In addition, IFN-α also did not show synergistic effect with TNF-α in apoptosis induction. These results suggest that HCC cells are resistant to growth regulation by IFN-α treatment, although p-STAT1 and ISGs with pro-apoptotic functions are robustly activated.

### U-STAT1 serves as a feedback loop to block the inhibitory effect of p-STAT1 on hepatocellular carcinoma cell growth

**Fig. 6:**
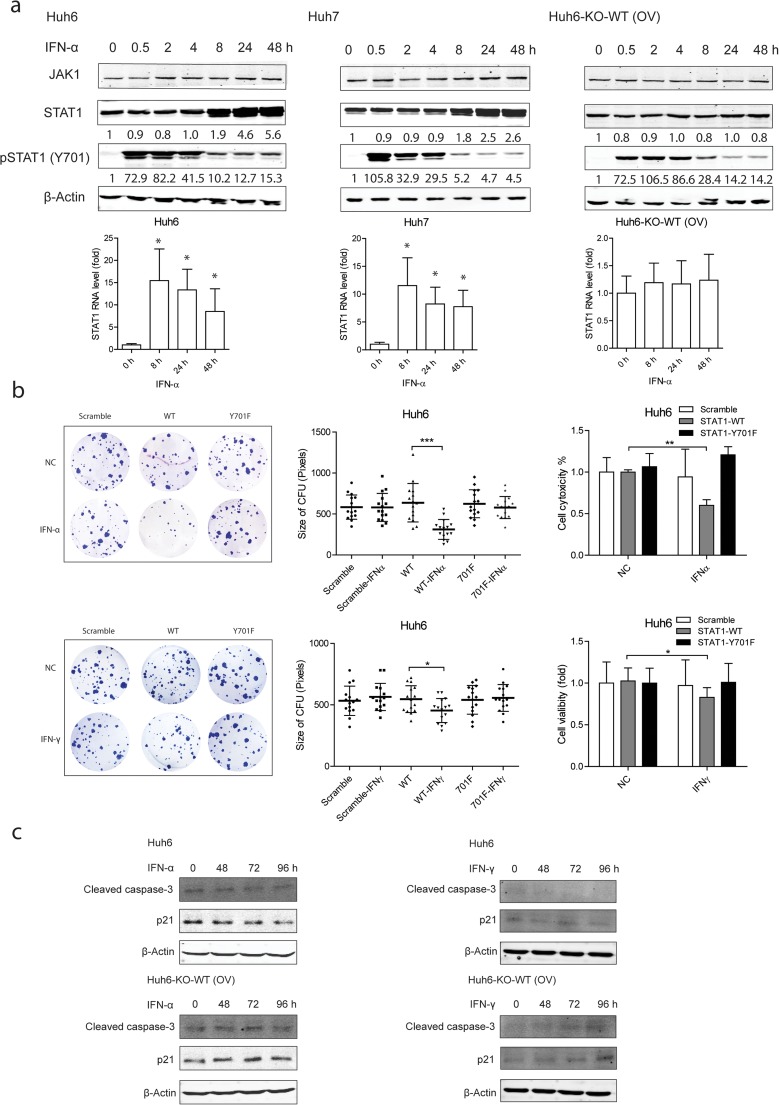
u-STAT1 works as a feedback loop in blocking p-STAT1 function. **a** IFN-α treatment induced u-STAT1 and p-STAT1 expression. Expressions of u-STAT1 and p-STAT1 were both induced in Huh7 and Huh6 cells by IFN-α but not in Huh6-KO-WT determined by western blot and qRT-PCR (mean ± SD, *n* = 4, *P < 0.05). **b** Attenuating u-STAT1 expression sensitized Huh6 cell to IFN treatment. Huh6-KO-WT and Huh6-KO-Y701F cells were treated with IFN-α (1000 IU/ml) or IFN-γ (1000 ng/ml). Decreased colony formation efficiency was found in Huh6-KO-WT and also cell growth inhibition but not in Huh6-KO-Y701F cells determined by MTT assay (mean ± SD, *n* = 3, **P < 0.01). **c** Blocking IFN-α-induced u-STAT1 expression sensitized HCC cells to apoptosis and cell cycle arrest. Cell lysates of Huh6 cells and Huh6-KO-WT treated with IFN-α (1000 IU/ml) or IFN-γ (1000 ng/ml) were collected for western blot analysis. β-actin served as loading control

To understand why HCC cells are insensitive to IFN-α treatment, we profiled the dynamic change of p-STAT1 and u-STAT1 expression. In fact, STAT1 is one of the most important ISGs. Both p-STAT1 and u-STAT1 were strongly induced by IFN-α. P-STAT1 peaked at 0.5 h after IFN-α treatment and thereafter decreased gradually, whereas u-STAT1 started to gradually increase 8 h post-treatment (Fig. 6a). The expression of JAK1 was not changed, which has been demonstrated to be inhibited by u-STAT1 [19]. We hypothesize these two forms may antagonize each other, and eventually deters the response to IFN-α treatment.

To dissect these complicated interactions, we artificially control STAT1 expression by genome modification. We exogenously expressed CMV promotor controlled WT (Huh6-KO-WT) or mutant (Y701F) (Huh6-KO-Y701F) STAT1 in STAT1 knockout Huh6 cells. Thus, STAT1 mRNA was constitutively expressed driven by the exogenous CMV promoter and therefore no longer be induced by IFN-α. Treatment of IFN-α activated p-STAT1, but the expression of u-STAT1 was no longer induced in these cells (Fig. 6a). As expected, ISG expression was strongly induced by IFN-α by re-expressing STAT1 in knockout cells, although no major effect on the basal expression of ISGs (Fig. S3). Importantly, both the colony formation and MTT assays show that blocking the induction of u-STAT1 expression sensitized Huh6-KO-WT cells to IFN-α/γ treatment. In contrast, this effect was not observed in Huh6-KO-Y701F, in which STAT1 cannot be phosphorylated, indicating the requirement of p-STAT1 activation for IFN-α/γ function (Fig. 6b). Even though STAT1 is important for sustaining the HCC cell growth, restoration of u-STAT1 expression in Huh6 knockout cells did not promote HCC growth (Fig. S4). Furthermore, by blocking u-STAT1 induction, both cleaved caspase-3 and p21 expression were stimulated in Huh6-KO-WT cells, but decreased in Huh6 cells (Fig. 6c). These results suggest that the induction of u-STAT1 as a feedback loop antagonizes the function of p-STAT1 and protects HCC cells from IFN-α treatment.

## Discussion

As the key component of IFN signaling, STAT1 have been reported with both pro- and anti-tumor functions during cancer development from clinical studies in cancer patients [8]. Deregulated expression of STAT1 has been observed in a variety of cancer types [31–34]. It is closely correlated to clinical behaviors of patients, either good or poor prognosis [16, 33]. In HCC, the expression of STAT1 has been reported to be lower in tumor tissues and is negatively associated with the histological grade [18]. However, we found that expression of STAT1 is higher in HCC tumor tissues in both our patients and other cohorts from online datasets, consisting of a large number of patients. Compared to the tumor-free tissues, we found higher levels of STAT1 is in the cytoplasm of HCC cells, whereas the levels in nuclear are comparable. The exact reasons accounting for the discrepancy between our results and the previous studies remain to be further investigated [18].

The phosphorylation status is essential for the functions of STAT1. In general, p-STAT1 is supposed to locate in nuclear, while u-STAT1 is considered predominately present in cytoplasm [27]. Surprisingly, we found that p-STAT1 is completely absent in our HCC tumor tissues and HCC cell lines, indicating that u-STAT1 is the dominant form located in both nuclear and cytoplasm. This is consistent with previous finding that u-STAT1 can shuttle between cytoplasm and nuclear and reinforces host defense against viral infection [29]. However, the expression levels of STAT1 in either nuclear or cytoplasm are not significantly related to survival in our patients.

Experimental studies in STAT1 knockout mouse have demonstrated a tumor suppressor function mainly through tumor intrinsic and extrinsic mechanisms [17, 35]. Cell cycle regulator, apoptosis inducers, and genes of immune system have been recognized as downstream targets of STAT1. However, several oncogenes have been reported to be regulated by STAT1, which are involved in promotion of tumor growth and invasiveness, suppression of immune surveillance, and induction of therapy resistance [8]. Thus, STAT1 plays multifaceted roles in cancer development. In HCC, we found that silencing u-STAT1 inhibits cell growth and arrests cell cycle, indicating u-STAT1 sustains the growth of HCC. Furthermore, ISGs recognized as IRDS were selectively regulated by u-STAT1, which may lead to resistance to DNA damage. These results are partially consistent with previous finding that u-STAT1 can protect tumor cells from apoptosis stimuli, radio- and chemotherapy [27, 34, 36, 37].

The classically active form of STAT1, p-STAT1, is strongly induced during immune response and rapidly regulates downstream gene expression. It has been demonstrated that p-STAT1 remarkably arrests tumor cell growth [23, 38, 39]. In line with this, we found that p-STAT1 inhibits HCC cell growth by arresting cell cycle and inducing cell apoptosis. ISGs with pro-apoptotic functions, such as FAS, TRAIL are only regulated by p-STAT1 but not u-STAT1. However, p-STAT1 is quickly dephosphorylated within only a few hours. u-STAT1, which is transcribed by p-STAT1, subsequently substitutes p-STAT1 expression and lasts for several days. Consequently, the anti-tumor effect of p-STAT1 is attenuated by the pro-tumor effect of u-STAT1. Thus, the function of STAT1 is highly dependent on its phosphorylation state, and p-STAT1 and u-STAT1 exert distinct functions.

IFNs have been widely explored for treating various malignancies [6]. However, IFN monotherapy has limited efficacy, although combination of IFNs with other tumoricidal therapies have been proven effective [40]. Systemic thermotherapy with IFNs for HCC has limited benefit on patient survival and in some instances is accompanied with significant toxicity [41], although antiviral therapy with IFNs might reduce the risk of virus infection in cancer patients [4]. Reasons for the clinical failure of IFNs likely include inherent biological mechanisms, changes in cell population, and institution of counter-regulatory pathways [42]. IFN signaling is generally considered to stimulate immune response, but it has also been reported to induce immunosuppression [9]. Different forms of STAT1, p-STAT1 and u-STAT1, have shown different transcription properties that contribute to the complexity of IFN signaling [9]. In our study, we have demonstrated that p-STAT1 and u-STAT1 play dual roles in HCC during IFN treatment. These results may explain the possible mechanisms of the ambiguous effects of IFNs in cancer treatment.

In summary, STAT1 is dominantly present as the form of u-STAT1 in HCC cells. The phosphorylation state deters the functions of STAT1 that u-STAT1 sustains but p-STAT1 inhibits HCC growth. Upon IFN treatment, the expression, phosphorylation, and localization of STAT1 are dynamically regulated and coordinately control the responsiveness to IFN treatment. Thus, these findings provide mechanistic insight on the role of STAT1 in HCC and provide scenario for future optimization of IFN treatment.

## Electronic supplementary material

ESM 1 (DOCX 58572 kb)
