# Supplementary material for: Dichotomal functions of phosphorylated and unphosphorylated STAT1 in hepatocellular carcinoma
Source: J Mol Med (Berl). 2018 Nov 19;97(1):77–88. doi: 10.1007/s00109-018-1717-7 (PMC6326978; doi:10.1007/s00109-018-1717-7)
Supplement: Supplementary file 1 — (DOCX 58572 kb) [file 109_2018_1717_MOESM1_ESM.docx]

**Supplementary information to**

**Dichotomal functions of phosphorylated and unphosphorylated STAT1 in hepatocellular carcinoma**

Buyun Ma, Kan Chen, Pengyu Liu, Meng Li, Jiaye Liu, Kostandinos Sideras, Katharina Biermann, Dave Sprengers, Wenshi Wang, Jan N.M. IJzermans, Wanlu Cao, Jaap Kwekkeboom, Maikel P. Peppelenbosch and Qiuwei Pan

**Supplementary Materials and Methods**

**Bioinformatics analysis of online datasets**

To analyze mRNA expression of STAT1 in HCC, the Oncomine microarray database (https://www.oncomine.org) was analyzed using the online tool. In the Gene Expression Omnibus (GEO) database , datasets of HCC gene expression were searched and analyzed (accession codes GSE14520). STAT1 mRNA expression was analyzed in identified cohorts by comparing expression levels in HCC tumors with tumor-free tissues. Moreover, survival data of 360 HCC cases in The Cancer Genome Atlas (TCGA) were available (https://portal.gdc.cancer.gov/projects/TCGA-LIHC).

**Immunohistochemistry**

Paraffin-embedded TMA slides were deparaffinized with xylene and rehydrated in graded alcohols (100%, 95%, 70%) for further immunohistochemistry staining. Slides were then washed with Phosphate Buffered Saline with Tween 20 (PBST) and boiled in citric acid buffer (pH6.0) for 20 min for antigen retrieval. Peroxidase was blocked by adding 3% H_2_O_2_ for 10 min at room temperature. The slides were incubated overnight with the primary antibody against STAT1 (rabbit polyclonal; sc-592) (1:300) and p-STAT1 (58D6; rabbit monoclonal; #9176) (1:150) at 4°C. After being rinsed in PBST, slides were incubated with second anti-rabbit IgG antibody conjugated with HRP for 1 h at room temperature. DAB solution (0.05% DAB, 0.0017% H_2_O_2_) was then prepared and added to the slides to visualize antibody binding. The reaction was stopped by washing with distilled water. Subsequently, hematoxylin were employed for background staining of tissue. Negative control staining was carried out by omitting the primary antibody.

Cytoplasmic and nuclear staining were scored separately. Percentages of cells with cytoplasmic or nuclear expression were scored as follows: low for 0-30%; moderate for 30-70%; high for > 70%. Scoring of expression intensity was performed as: grade 1 for weak; grade 2 for moderate; grade 3 for strong. A final immune-reactivity score (IRS) was obtained for each case by multiplying the percentage and the intensity values, ranging from low, moderate and high. The scorings were done by two investigators.

**Cell culture and reagents**

Seven different human hepatoma cell lines (Huh7, Huh6, PLC, snu398, snu449, snu182, HepG2) were cultured in Dulbecco’s modified Eagle’s medium (DMEM; Lonza). Media were supplemented with 10% (v/v), fetal bovine serum (FBS) (Hyclone Technologies), 100 units/mL of penicillin and 100 μg/mL of streptomycin. All the cells were incubated at 37°C in a humidified atmosphere containing 5% CO_2_. All the cell lines were a kind gift from Dr. Ron Smits (department of Gastroenterology and Hepatology, Erasmus MC-University Medical Center) [1] and confirmed mycoplasma-free and their STR genotyping was analyzed at the Department of Pathology, Erasmus Medical Center Rotterdam.

**Generation of STAT1 knockout cells using LentiCRISPR/Cas9 system**

The sgRNA (TCCCATTACAGGCTCAGTCG) targeting *STAT1* was designed by online tool “MIT CRISPR Design” (http://crispr.mit.edu/) and cloned into the lentiviral backbone vector lentiCRISPR v2 (Addgene). To produce lentivirus, HEK293T cells were transfected with 0.6 µg of pMD.2G, 1.5 µg of psPAX2, and 2 µg of lentiCRISPR v2 in a 6-well plate. Lentivirus-containing culture supernatants were collected and filtered through a 0.45 µm filter. Cells were then infected with lentivirus for two days and selected using 3.0 µg/ml puromycin (Sigma-Aldrich). From stably transduced cell lines, single cells were sorted by FACS, and genomic DNA was isolated using Wizard® Genomic DNA Purification Kit (Promega) to identify the introduced mutations (Figure. S2). To further validate the knockout effect, western blot was performed to detect the expression of STAT1 at protein level.

**Colony formation assay**

Cells were trypsinized, harvested and suspended in culture medium. After quantified through counting, 2 × 10^3^ cells were seeded into 6-well plates and the medium was refreshed for every four days. After two weeks culture, formed colonies were washed with PBS and fixed by 70% ethanol. Followed by counterstaining with crystal violet and washed with PBS, colony sizes were measured microscopically through digital image analysis.

**MTT assay**

Cells were trypsinized and seeded in a 96-well plate at a concentration of 1 × 10^3^ cells/well. After overnight incubation, cells were treated with IFN-α (1000, 5000, 10000 IU/ml) for one week and the medium was refreshed for one time. Cells were then incubated with 0.5 mg/ml MTT (Sigma-Aldrich) for 4 h. After discarding the cell supernatant, 150 µl DMSO was added followed 10 min shaking. The absorbance was determined using enzyme mark instrument at the wavelength of 490 nm. The formula: (Absorbance _treated cells_-Absorbance _DMSO_)/(Absorbance _negative control_-Absorbance _DMSO_) was used to analyze the cell viability.

**Cell cycle analysis**

Cells (5 × 10^5^/well) were plated in six-well plates and allowed to attach overnight. When the cell confluence reached 60% to 80%, cells were trypsinized and washed with PBS for two times and then fixed in cold 70% ethanol overnight at 4°C. The cells were then washed twice with PBS and incubated with 50 µl RNase (100 μg/ml ) at 37°C for 30 min, and then 250 µl propidium iodide (PI) (50 μg/ml) was added and cells were incubated at room temperature for 5 min. The samples were analyzed immediately by FACS. Cell cycle was analyzed by FlowJo software.

**Cell apoptosis analysis**

Cell apoptosis analysis was performed by staining cells with annexin V-FITC (BD Pharmingen) and PI. Cells (5 × 10^5^/well) were seeded into six-well plates and incubated at 37°C in 5% CO_2_ overnight, then cells were treated with IFN-α (Thermo Scientific, the Netherlands) (1000 IU/ml), TNF-α (Peprotech, USA) (20 ng/ml) or the combination. After 72 h, all of the cells were trypsinized and resuspended in annexin-binding buffer (BD Pharmingen) and stained with Alexa Fluor 488 Annexin V and PI at room temperature for 15 min. Detection of apoptosis was performed by FACS and the results were analyzed by FlowJo software.

**Western blot assay**

Laemmli sample buffer containing 0.1 M DTT (freshly made) was used to lyse the cells. Then, cell lysates were denaturalized by heating 5-10 min at 95°C followed by loading onto a 10-15% sodium dodecyl sulfate-polyacrylamide SDS gel and separated by electrophoresis (SDS-PAGE). After 90 min running in 120 V, proteins were electrophoretically transferred onto a polyvinylidene difluoride (PVDF) membrane (Invitrogen) for 1.5 h with an electric current of 250 mA. Subsequently, the membrane was blocked with blocking buffer (Li-COR, Lincoln, USA) mixed with PBST in ratio of 1:1. And then followed by overnight incubation with rabbit anti-STAT1, anti-p-STAT1 (Y701) (1:1000) antibody at 4°C. Membrane was washed 3 times with PBST, which was followed by incubation for 1 h with anti-rabbit or anti-mouse IRDye-conjugated secondary antibodies (Li-COR, Lincoln, USA) (1:5000) at room temperature. Blots were assayed for actin content as standardization of sample loading, and scanned and quantified by odyssey infrared imaging (Li-COR, Lincoln, USA). The results were analyzed with Odyssey 3.0 software.

**
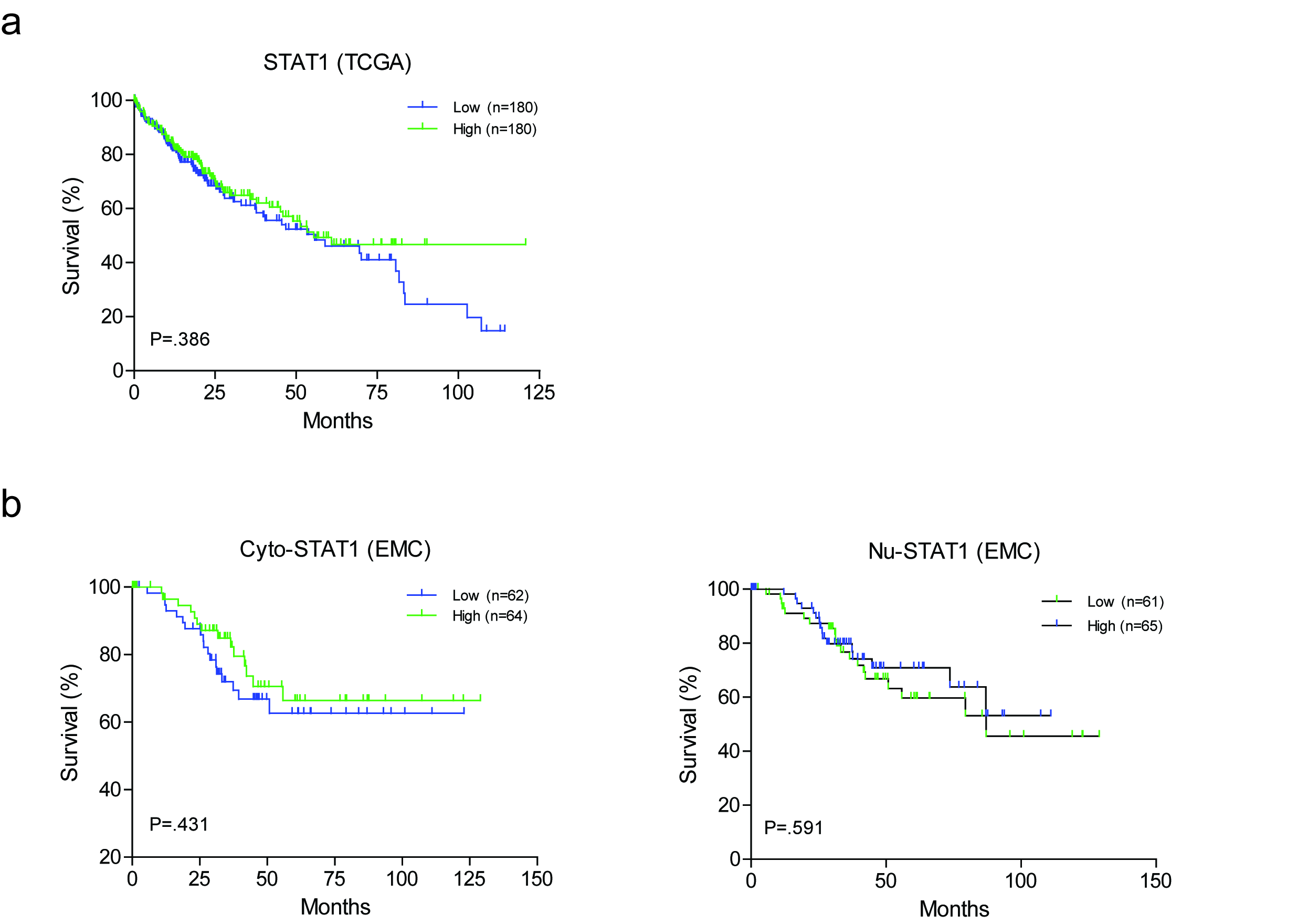
**

**Figure S1. Survival analysis of HCC patients.**

a Kaplan Meier analysis of HCC patients from the TCGA cohort. Expression of STAT1 is not strongly associated with patient outcome (n=360, p=0.386). b Kaplan Meier analysis of the EMC patient cohort. Both cytoplasm (n=126, p=0.431) and nuclear expression (n=126, p=0.591) were analyzed for patient survival outcome. No significant correlation with patient outcome was found.


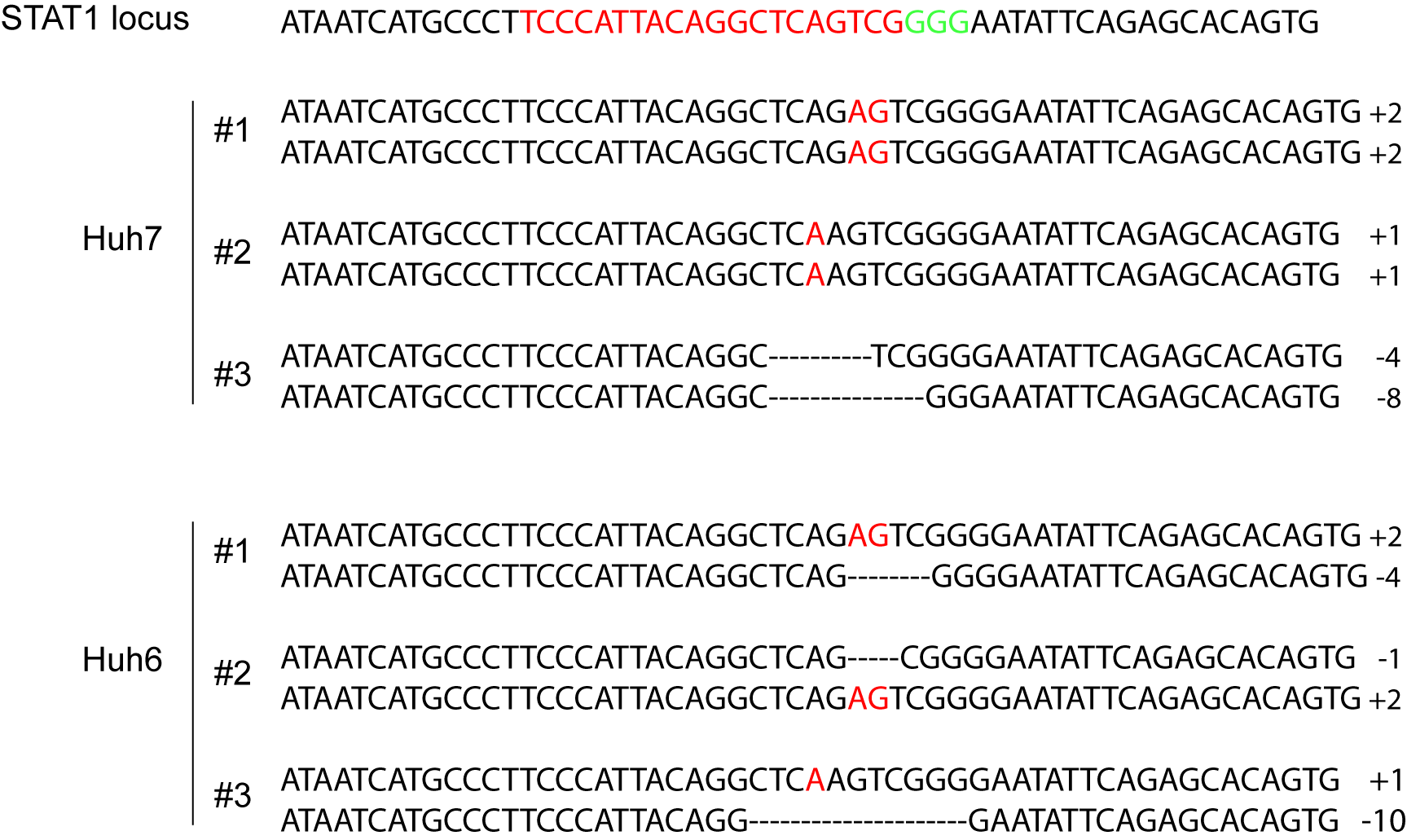


**Figure S2. Genome sequencing of STAT1 knockout cell clones.**

The sequence locus of STAT1, red labeled as the sgRNA targeting site and green labeled as the PAM sequence. All the clones show frameshift mutation with nucleotides deletion (dash line) or insertion (red).

**Figure S3. Expression of ISGs are restored by STAT1 overexpression.**

a STAT1 re-expression does not further induce the expression of ISGs (mean ± SD, n=3, two replicates *p<0.05). b STAT1 expression restores the function of IFN-α in inducing ISG expression. Huh6 cells transduced with lentiviral vector expressing STAT1 were treated with IFN-α for 24 h (mean ± SD, n=3, two replicates *p<0.05), NC=negative control (Huh6 transfected with empty lenti-vector)

**Figure S4. STAT1 did not promote HCC cell growth**

Huh6-KO-WT and Huh6-KO-Y701F cells were grown for one week. Cell growth was determined by MTT assay (mean ± SD, n=3, two biological replicates for each independent experiment).

**Table S1. Human qPCR primer sequences**

|  | 5' FORWARD | 3' REVERSE |
| --- | --- | --- |
| GAPDH | GTCTCCTCTGACTTCAACAGCG | ACCACCCTGTTGCTGTAGCCAA |
| IFIT1 | GCCTTGCTGAAGTGTGGAGGAA | ATCCAGGCGATAGGCAGAGATC |
| IFIT3 | CCTGGAATGCTTACGGCAAGCT | GAGCATCTGAGAGTCTGCCCAA |
| IFI27 | CGTCCTCCATAGCAGCCAAGAT | ACCCAATGGAGCCCAGGATGAA |
| ISG15 | CTCTGAGCATCCTGGTGAGGAA | AAGGTCAGCCAGAACAGGTCGT |
| OAS1 | AGGAAAGGTGCTTCCGAGGTAG | GGACTGAGGAAGACAACCAGGT |
| MX1 | GGCTGTTTACCAGACTCCGACA | CACAAAGCCTGGCAGCTCTCTA |
| IRF1 | GAGGAGGTGAAAGACCAGAGCA | TAGCATCTCGGCTGGACTTCGA |
| STAT1 | ATGGCAGTCTGGCGGCTGAATT | CCAAACCAGGCTGGCACAATTG |

**Table S2. Patient characteristics according to HCC specific mortality.**

| **Variable** |  | **HR(95%CL)** | ***P*** |
| --- | --- | --- | --- |
| Age |  | 0,735 (0,287-1,884) | 0,522 |
| AFP (>200)*** | | 6,808 (2,207-16,797) | 0,000 |
| Tumor Size | | 3,298 (0,967-11,244) | 0,057 |
| Fibrosis |  | 1,341 (0,392-4,582) | 0,640 |
| Cirrhosis |  | 1,238 (0,436-3,516) | 0,688 |
| Vascular invasion | | 1,524 (0,644-3,604) | 0,338 |
| HBV positive | | 0,003 (0,000-1,659E+63) | 0,941 |
| HCV positive | | 0,006 (0,000-3,011E+63) | 0,947 |
| Viral hepatitis | | 305,841 (0,000-1,505E+68) | 0,941 |
| Differentiation* | | 0,242 (0,070-0,831) | 0,024 |
| Gender |  | 1,468 (0,590-3,651) | 0,409 |

**Table S3. Patient characteristics according to cytoplasmic STAT1 expression**

| **Variable** |  | **HR(95%CL)** | ***P*** |
| --- | --- | --- | --- |
| Age |  | 0,981 (0,466-2,068) | 0,960 |
| AFP (>200) | | 0,959 (0,314-3,006) | 0,959 |
| Tumor Size | | 1,707 (0,632-4,607) | 0,291 |
| Fibrosis |  | 1,878 (0,543-6,494) | 0,319 |
| Cirrhosis |  | 0,887 (0,371-2,122) | 0,788 |
| Vascular invasion | | 1,261 (0,576-2,762) | 0,561 |
| HBV positive | | 0,855 (0,361-2,026) | 0,721 |
| HCV positive | | 0,422 (0,153-1,167) | 0,097 |
| Differentiation | | 0,541 (0,191-1,533) | 0,248 |
| Gender |  | 0,850 (0,390-1,852) | 0,683 |

**Table S4. Patient characteristics according to nuclear STAT1 expression**

| **Variable** |  | **HR(95%CL)** | ***P*** |
| --- | --- | --- | --- |
| Age** |  | 0,372 (0,178-0,778) | 0,009 |
| AFP (>200) | | 2,401 (0,967-5,964) | 0,059 |
| Tumor Size | | 0,976 (0,468-2,032) | 0,947 |
| Fibrosis |  | 0,606 (0,202-1,814) | 0,371 |
| Cirrhosis |  | 1,235 (0,510-2,991) | 0,640 |
| Vascular invasion | | 0,632 (0,303-1,319) | 0,222 |
| HBV positive | | 0,308 (0,025-3,749) | 0,356 |
| HCV positive | | 0,602 (0,059-6,142) | 0,668 |
| Viral hepatitis | | 1,994 (0,156-25,487) | 0,595 |
| Differentiation | | 2,662 (0,960-7,369) | 0,060 |
| Gender |  | 0,717 (0,363-1,415) | 0,338 |

**References**

1. Wang W, Xu L, Liu P, Jairam K, Yin Y, Chen K, Sprengers D, Peppelenbosch MP, Pan Q, Smits R (2016) Blocking Wnt Secretion Reduces Growth of Hepatocellular Carcinoma Cell Lines Mostly Independent of beta-Catenin Signaling. Neoplasia 18: 711-723.
